# Elevation of autophagy markers in Sjögren syndrome dry eye

**DOI:** 10.1038/s41598-017-17128-0

**Published:** 2017-12-08

**Authors:** Yong-Soo Byun, Hyun Jung Lee, Soojung Shin, So-Hyang Chung

**Affiliations:** 1 Department of Ophthalmology and Visual Science, Seoul St. Mary’s Hospital, Catholic University of Korea, College of Medicine, Seoul, Republic of Korea; 2 Catholic Institute for Visual Science, Catholic University of Korea, College of Medicine, Seoul, Republic of Korea

## Abstract

Autophagy is known to be implicated in the pathogenesis of Sjögren syndrome (SS), but evidences are limited. We aimed to examine the levels of autophagy markers in tear film and conjunctival epithelial cells from SS dry eye patients, and analyze their correlations with clinical features. Patients with SS dry eye exhibited lower Schirmer values, lower tear breakup time, and higher ocular staining scores. In tears, ATG5 and LC3B-II/I levels were significantly higher in SS dry eye. ATG5 and LC3B-II mRNA in the conjunctiva were also elevated in SS dry eye compared with non-SS dry eye. The immunostaining of conjunctival epithelium showed a punctate pattern of ATG5 and LC3B-II in SS dry eye. These staining patterns were also observed in the lacrimal gland of SS animal models. ATG5 levels in tears and the conjunctival epithelium strongly correlated with ocular staining scores, and one month of topical corticosteroid treatment reduced both ATG5 and LC3B-II/I levels in tear film and the conjunctival epithelium of patients with SS dry eye. Our results suggest that autophagy is enhanced or dysregulated in SS and autophagy markers may be serve as both diagnostic and therapeutic biomarkers in SS dry eye.

## Introduction

Dry eye (DE) syndrome is a multifactorial disease characterized by tear film instability and ocular surface damage, which cause foreign body sensation, discomfort and visual disturbances^1^. Among various etiologies, Sjögren syndrome (SS) is a major cause of aqueous deficient type DE^1^. SS is a systemic autoimmune disease that affects the entire body, mainly the exocrine glands^2^. The characteristics of SS is the lymphocytic infiltration of the exocrine glands and mucosal epithelia, causing dry eye and dry mouth^3,4^. The pathogenic mechanisms of SS is assumed to lead to more severe dry eye manifestations compared to non-SS DE^5–7^.

Numerous studies over the past decade extended our understanding of the pathologic mechanisms of SS. Autophagy has been suggested to have a role in the pathogenesis of SS, although data to support this role are limited^8–10^. Recent data demonstrated that enhanced autophagy and apoptosis are involved in the Ro/SAA and La/SSB redistribution in secretory epithelial cells of the salivary gland^9,11,12^. At the cellular level, the local secretion of autoantibodies follows the re-localization of the autoantigens to the cell surface^13,14^. Autophagy is a self-eating cellular process to maintain cellular homeostasis via lysosome-mediated degradation and recycling of cytoplasmic components and organelles. In addition to its classic role in response to cellular stress, autophagy has been implicated in the pathogenesis of autoimmune diseases^15–17^. Autophagy regulates various immune processes, such as antigen presentation, pathogen removal, the survival immune cells, and inflammation^17^. Moreover, recent studies showed that polymorphisms in autophagy-related genes might attribute to susceptibility of systemic lupus erythematosus and Crohn’s disease^18,19^.

During autophagy, a portion of the cytoplasm and several proteins are incorporated in the autophagosome, a key structure with double layer membranes for intracellular degradation. Among these proteins, ATG5 and the LC3B-II/I ratio are used as typical markers of autophagy. ATG5 (autophagy related gene 5), which conjugates with ATG12, is specifically required for the maturation of the autophagic membrane, and ATG5 deletion plays a role in autophagy-mediated salivary homeostatic control in SS^8^. A cytosolic form of LC3 (LC3-I) is conjugated to phosphatidylethanolamine to form LC3-phosphatidylethanolamine conjugate (LC3-II), which is recruited to the autophagosomal membranes^20^. The aim of this study was to investigate these autophagy markers in tear film and conjunctival epithelial cells from SS DE and non-SS DE patients as well as to analyze their correlations with clinical features for determining the implications of autophagy dysregulation in SS DE.

## Results

### Patient Demographics

The demographics and ocular surface parameters of subjects are listed in Table 1. Schirmer I value and TBUT were significantly lower and the conjunctival staining scores were significantly higher in SS DE than non-SS DE. Age, OSDI, and corneal staining scores did not significantly differ between SS DE and non-SS DE.

**Table 1:** Clinical Parameters in Sjögren Syndrome Dry Eye, non-Sjögren Syndrome Dry Eye, and Normal Controls.

Characteristics	SS DE	non-SS DE	Normal control	*P* value^a^
No. of patients (eyes)	40 (78)	24 (48)	16 (25)	
Age (y)	53.80 ± 11.77 (28 to 78)	50.13 ± 14.80 (18 to 80)	53.43 ± 16.76 (25 to 78)	0.3602
OSDI (0–100)	47.57 ± 26.19 (2 to 100)	37.59 ± 17.94 (3.5 to 70.45)	6.77 ± 13.95 (0 to 42.5)	0.0938
Schirmer I value (mm)	2.72 ± 1.88 (0 to 8)	5.81 ± 2.21 (2 to 10)	11.46 ± 3.66 (2 to 15)	<0.0001
TBUT (s)	2.82 ± 2.30 (0 to 10)	4.63 ± 2.38 (2 to 10)	7.58 ± 1.90 (4 to 10)	<0.0001
Corneal staining score (0–6)	2.22 ± 1.47 (0 to 6)	1.73 ± 1.01 (0 to 4)	0	0.0579
Conjunctival staining score (0–6)	2.12 ± 1.34 (0 to 5)	0.77 ± 0.95 (0 to 3)	0	<0.0001

SS = Sjögren Syndrome, DE = dry eye, OSDI = ocular surface disease index; TBUT = tear film break-up time; OSS = ocular surface staining.

^a^comparison between SS DE and non-SS DE.

### Autophagy Marker Expression in Tear and Conjunctival Epithelial Cells

**Figure 1:**
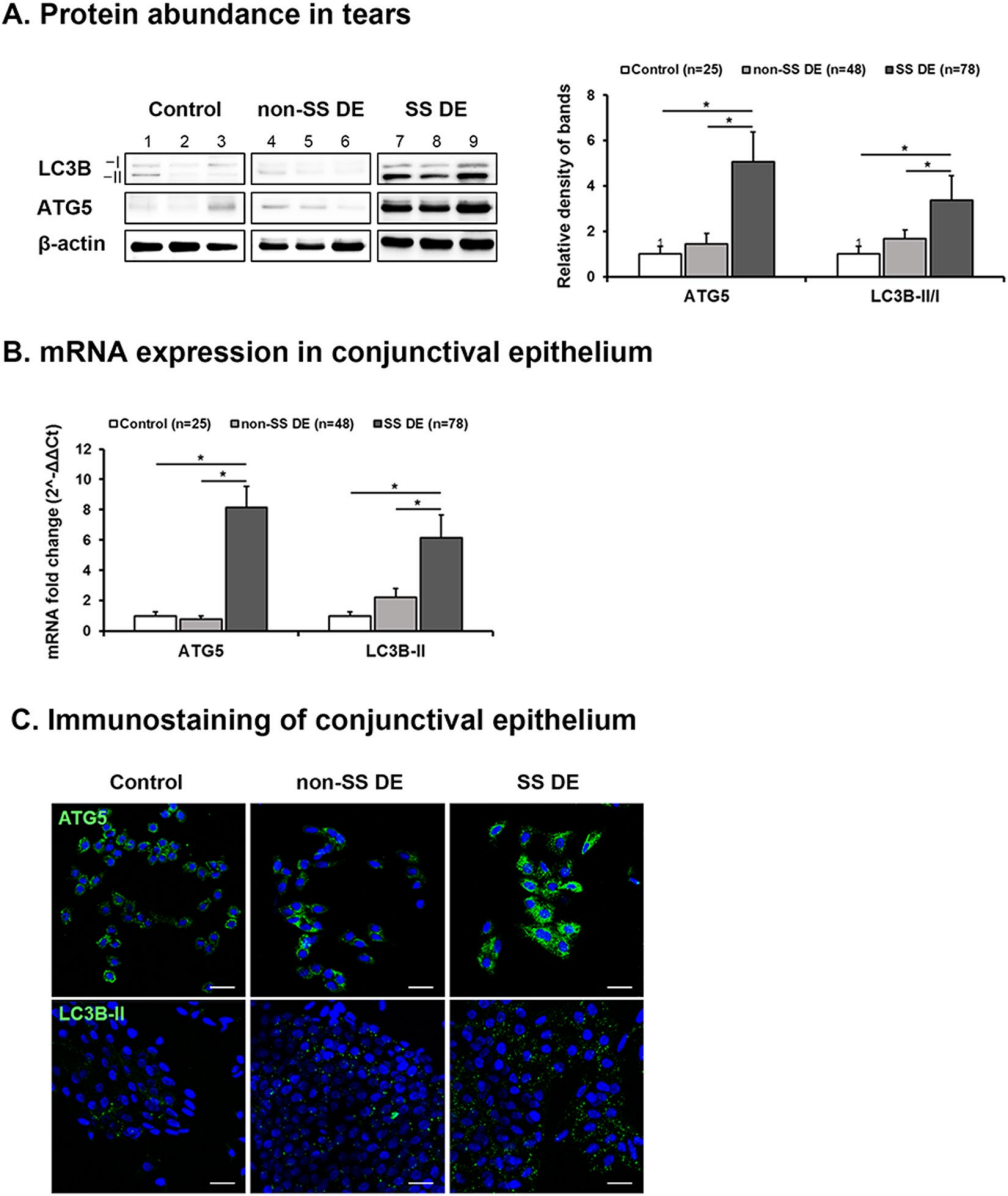
Expression of Autophagy Markers ATG5 and LC3B in Tears and Conjunctival Impression Cytology Specimens from Controls, Patients with non-Sjögren Syndrome Dry Eye, and Patients with Sjögren Syndrome Dry Eye. (**A**). Representative Western blot images and relative densitometry data on ATG5 protein expression and the LC3B-II/I conversion ratio in tears demonstrating increases in patients with Sjögren Syndrome (SS) dry eye (DE) compared to patients with non-SS DE and controls. The protein expressions of ATG5, LC3B-I, LC3B-II and β-actin were developed from the same gel in western blot. (**B**). The ATG5 and LC3B-II mRNA expression levels in the conjunctiva as measured by real time PCR demonstrating increases in patients with SS DE compared to patients with non-SS DE and controls. (**C**). Representative confocal images of immunostained conjunctiva from impression cytology specimens showing that the punctate cytoplasmic staining patterns of ATG5 and LC3B-II indicative of autophagy were enhanced in patients with SS DE. *P < 0.05. Scale bar = 20 μm.

Western blot analysis of tears revealed significant increases in ATG5 protein and the LC3B-II/I conversion ratio in SS DE compared to non-SS DE and controls. The relative densitometry results showed that the levels of ATG5 (vs β-actin) and the LC3B-II/I ratio in tears were higher in patients with SS DE (5.06 ± 1.31 and 3.37 ± 1.08) than in patients with non-SS DE (1.44 ± 0.46 and 1.67 ± 0.40) and healthy controls (1.00 ± 0.35 and 1.00 ± 0.35) (*P* = 0.0001) (Fig. 1A). The mRNA levels of ATG5 and LC3B-II in the conjunctiva were also upregulated in patients with SS DE (8.15 ± 1.38- and 6.14 ± 1.51-fold change), but not in patients with non-SS DE (0.80 ± 0.17- and 2.21 ± 0.61-fold change), compared to healthy controls (Fig. 1B, all *P* < 0.0001). The expression levels of autophagy markers in tears and conjunctival impression cytology specimens did not significantly differ between non-SS DE and controls. The immunofluorescent staining of conjunctival impression cytology specimens identified a punctate cytoplasmic staining pattern of ATG5, indicative of autophagy, in SS DE, whereas ATG5 exhibited diffuse staining in samples from non-SS DE and controls (Fig. 1C). Furthermore, the number of punctate staining foci of LC3B-II was also notably increased in SS DE but not in non-SS DE and controls. Overall, autophagy marker expression was upregulated in tears and the conjunctiva of SS DE, suggesting that the dysregulation of autophagy may be implicated in the pathogenesis of SS DE.

### Autophagy Marker Expression in the Lacrimal Glands of Animal Models

**Figure 2:**
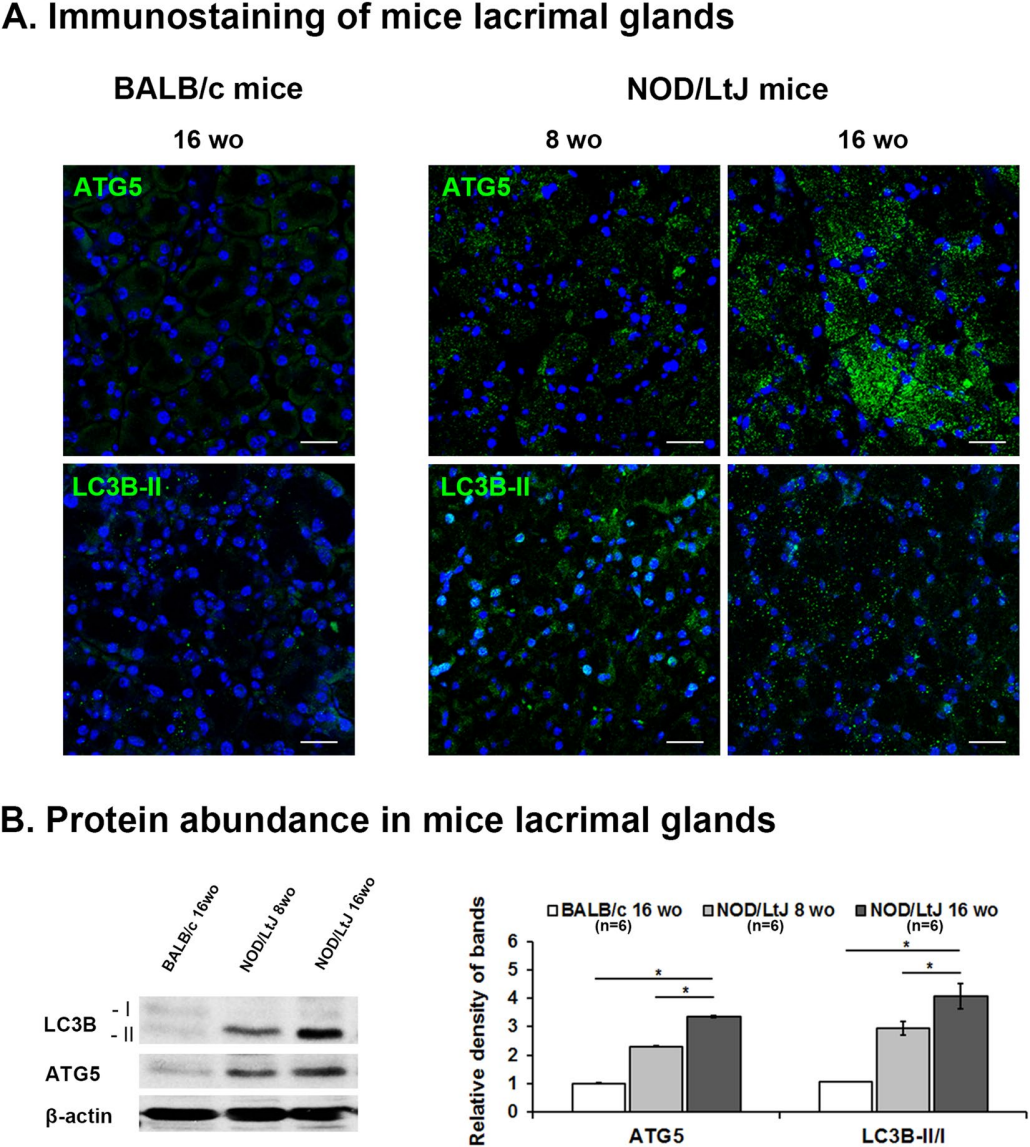
Immunofluorescent Staining of Autophagy Markers (ATG5 and LC3B-II) in the Lacrimal Glands of Sjögren Syndrome Animal Models (NOD/LtJ mice). (**A**) Representative immunofluorescent photos demonstrating cytoplasmic punctate staining patterns of ATG5 and LC3B-II on the lacrimal gland developed prominently at 16 weeks of gestational age in NOD/LtJ mice. Those markers were not prominent at 8-week-old NOD/LtJ mice. Lacrimal glands of 16-week-old BALB/c mice were used as a control. DAPI (blue) was used for nuclear counterstaining. Scale bar = 20 μm. (n = 6). (**B**) Western blotting and relative densitometry graph demonstrates ATG5 protein expression and the LC3B-II/I conversion ratio from lysates of 8− and 16-week-old NOD/LtJ, and 16-week-old BALB/c mice. Both markers were significantly higher at 16 weeks than 8 weeks of gestational age in NOD/LtJ mice (n = 6). The protein expressions of ATG5, LC3B-I, LC3B-II and β-actin were developed from the same gel in western blot. *P < 0.0001.

In the lacrimal glands of non-obese diabetic (NOD)/LtJ mice, punctate cytoplasmic staining patterns of ATG5 and LC3B-II were observed at 8 weeks of gestational age and developed prominently in lacrimal gland at 16 weeks of gestational age, compared to 16-week-old BALB/c normal mice (Fig. 2A). Correspondingly, western blot analysis from lysates of lacrimal gland showed higher levels of ATG5 and LC3B-II/I in 16-week-old NOD/LtJ mice (3.36 ± 0.04 and 4.07 ± 0.43 versus BALB/c normal mice) than in 8-week-old NOD/LtJ (2.30 ± 0.02 and 2.94 ± 0.22 versus BALB/c normal mice), respectively (Fig. 2B). Based on these findings in the SS animal model, we speculate that autophagy might be induced in the lacrimal glands of patients with SS DE.

### Correlations between Autophagy Markers and Clinical Parameters in SS DE

Because autophagy markers were significantly upregulated in SS DE but not in non-SS DE, we next investigated the correlation between autophagy markers and clinical parameters in patients with SS DE (Table 2). ATG5 expression strongly correlated with corneal and conjunctival staining scores in patients with SS DE, which indicates that ATG5 might be used as a biomarker in SS DE. Additionally, LC3B-II/I conversion ratio was not significantly correlated with any clinical parameters in this study.

**Table 2:** Pearson’s Correlation Coefficient Data between ATG5 and Clinical Parameters in Sjögren Syndrome Dry Eye (n = 78).

	r	95% confidence interval	*P* value
ATG5 (tear)			
vs. OSDI	0.1003	−0.2313 to 0.4110	0.5547
vs. Schirmer	−0.2781	−0.5596 to 0.0608	0.1057
vs. TBUT	0.0541	−0.2704 to 0.3675	0.7471
vs. corneal stain	0.4810	0.1951 to 0.6916	0.0019*
vs. conjunctival stain	0.5917	0.3354 to 0.7664	<0.0001*
ATG5 (IC)			
vs. OSDI	−0.4450	−0.7355 to −0.0164	0.0432*
vs. Schirmer	0.2439	−0.1982 to 0.6035	0.2741
vs. TBUT	0.0827	−0.3512 to 0.4875	0.7142
vs. corneal stain	0.7371	0.4576 to 0.8840	<0.0001*
vs. conjunctival stain	0.4604	0.04801 to 0.7387	0.0311*

OSDI = ocular surface disease index; TBUT = tear film break-up time; OSS = ocular surface stain.

### The Outcome of Topical Corticosteroid Treatment on Ocular Surface Parameters and the Level of Autophagy Markers in SS DE

**Figure 3:**
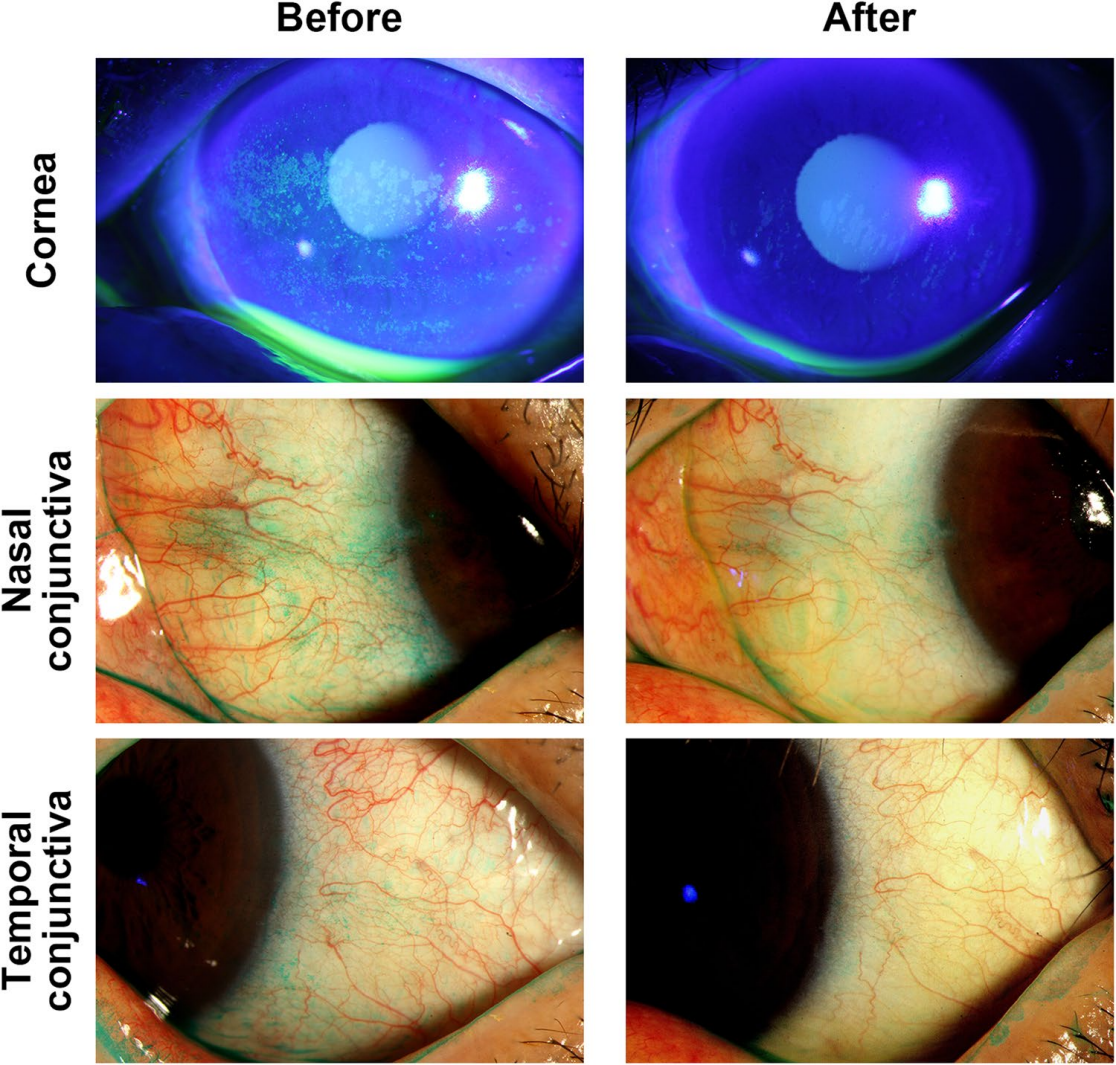
The Representative Images of Ocular Surface Staining Before and After Topical Corticosteroid Treatment in Sjögren Syndrome Dry Eye Patient. In this patient, the ocular surface staining scores, which were 5, 3, and 2 in cornea, nasal, and temporal conjunctiva before treatment, reduced to 3, 1, and 0 after 1-month topical corticosteroid treatment, respectively. Corneal and conjunctival staining was performed using fluorescein and lissamine green dye.

**Figure 4:**
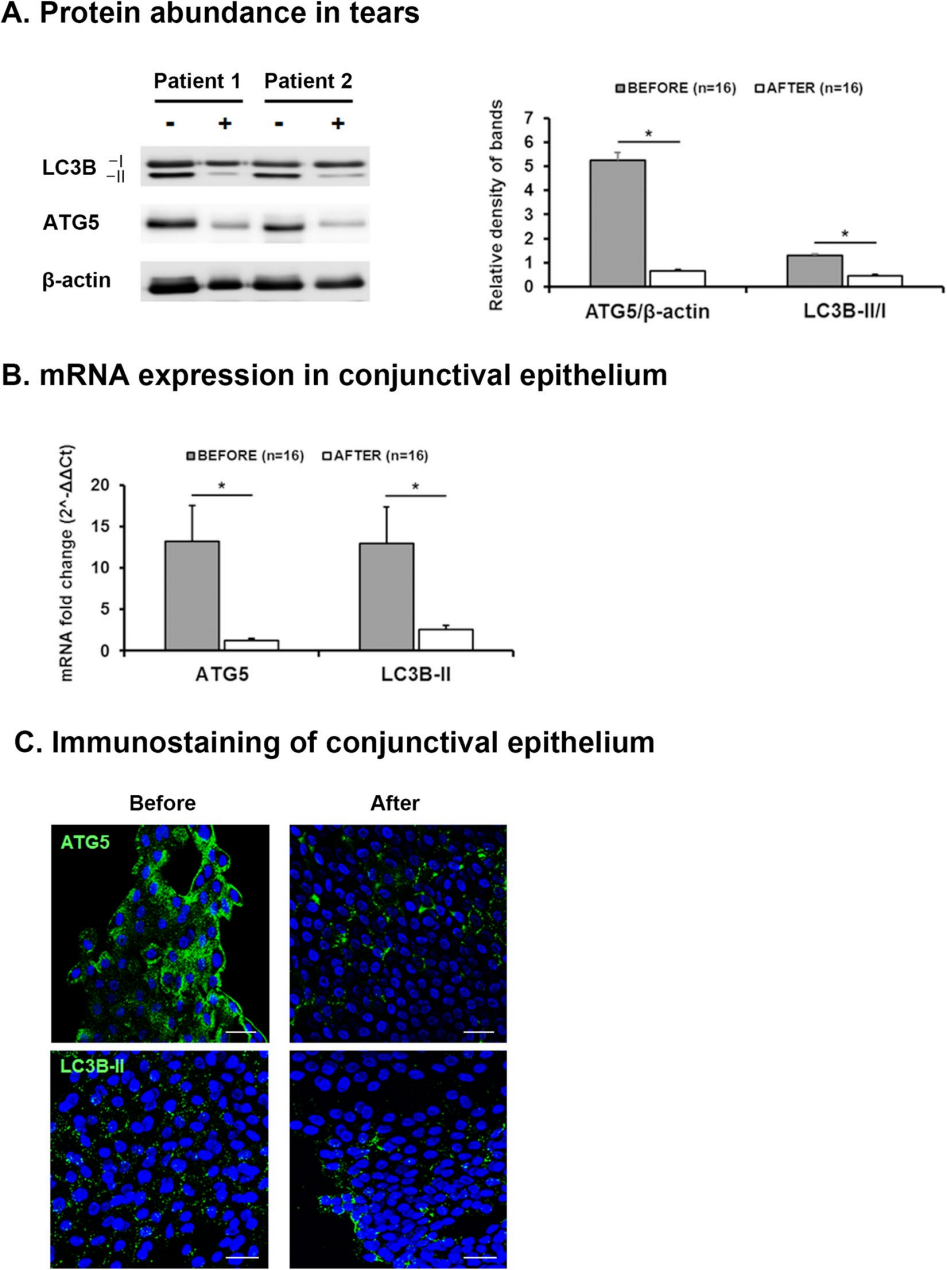
Changes in Autophagy Markers after Topical Corticosteroid Treatment. The expression levels of the autophagy markers ATG5 and LC3B-II/I in tears and conjunctival impression cytology specimens from 16 eyes of 16 patients with Sjögren syndrome (SS) Dry Eye (DE) before and after corticosteroid treatment. (**A**) Representative Western blot image of ATG5 and LC3B-II/I conversion in tears showing reductions in patients with SS DE after corticosteroid treatment. Relative densitometry of ATG5 (vs β- actin) and the LC3B-II/I ratio in tears showing significant decrease in patients with SS DE after corticosteroid treatment. The protein expressions of ATG5, LC3B-I, LC3B-II and β-actin were developed from the same gel in western blot. (**B**) ATG5 and LC3B-II mRNA expression level in the conjunctiva measured by real time PCR showing significant reductions in patients with SS DE. (**C**) The cytoplasmic punctate staining patterns of ATG5 and LC3B-II indicative of autophagy were markedly attenuated in the immune-stained conjunctiva impression cytology specimens of patients with SS DE after corticosteroid. *P < 0.05. Scale bar = 20 μm.

After administering topical corticosteroid to SS DE, we evaluated the changes in autophagy marker expression and clinical parameters. One month of topical corticosteroid treatment with artificial tears significantly improved the OSDI score, Schirmer I value, TBUT, corneal and conjunctival staining scores in SS DE (Table 3 and Fig. 3). Accordingly, representative Western blot images demonstrated reductions in the ATG5 and LC3B-II/I ratio in tears after topical corticosteroid treatment (Fig. 4A). The relative densitometry results showed that ATG5 expression (vs β-actin) and the LC3B-II/I ratio in tears significantly decreased after corticosteroid treatment (0.66 ± 0.05 and 0.84 ± 0.08) compared to the respective values before treatment (5.24 ± 0.32 and 1.29 ± 0.07) in SS DE (*P* < 0.0001 and *P* = 0.001, respectively). The mRNA levels of ATG5 and LC3B-II (fold) were significantly decreased from 13.16 ± 4.36 and 12.93 ± 4.44 to 1.23 ± 0.27 and 2.57 ± 0.48 (respectively, *P* = 0.00573 and *P* = 0.01586) (Fig. 4B). In addition, representative immunostaining pictures demonstrated that the punctate staining patterns of autophagy markers in the conjunctiva were also normalized in SS DE after corticosteroid treatment (Fig. 4C).

**Table 3:** Changes in Clinical Parameters after Topical Corticosteroid Treatment in 16 patients with Sjögren Syndrome Dry Eye.

	Before treatment (n = 16)	After treatment (n = 16)	*P* value
OSDI score (0–100)	50.19 ± 19.17 (15 to 80.5)	39.80 ± 17.89 (22.5 to 80)	0.0411
Schirmer value (mm)	2.34 ± 1.66 (0 to 6)	3.78 ± 2.41 (2 to 12)	0.0088
TBUT (s)	2.16 ± 2.22 (0 to 8)	3.84 ± 1.90 (2 to 8)	0.0007
Corneal staining score (0–6)	2.78 ± 1.10 (0 to 6)	1.59 ± 1.21 (0 to 4)	<0.0001
Conjunctival staining score (0–6)	2.13 ± 1.90 (0 to 5)	1.06 ± 1.70 (0 to 6)	0.0018

Abbreviation; OSDI = ocular surface disease index; TBUT = tear film break-up time.

## DISCUSSION

In the current study, we revealed that the ATG5 level and LC3B-II/I ratio in tears and mRNA expression levels in conjunctival epithelial cells significantly increased in SS DE. Our findings constitute first evidence to suggest that the autophagy markers were upregulated in SS DE. To date, limited studies have examined the role of autophagy in SS and the exact mechanisms linking autophagy to SS pathogenesis are not established. Previous studies reported that autophagy plays important roles in redistributing the auto-antigens Ro/SSA and La/SSB in salivary gland epithelial cells^9^ or maintaining the homeostasis of salivary acinar cells^8^. Autophagy dysregulation has been reported in various autoimmune and auto-inflammatory diseases. The promotion of the presentation of cytosolic antigens by major histocompatibility complex (MHC) class II and the control of T lymphocyte homeostasis enhance autophagic reaction, and type 1 T helper cells cytokines and specific serum autoantibodies may also play a role in this reaction^21–23^. ATG5 has been observed to be upregulated in patients with SLE, and complement-inactivated serum from patients significantly activated autophagy^24^. A more recent study directly investigating autophagy in T cells indicated that autophagy was increased in lupus, which promoted the survival of autoimmune T cells^25^. Furthermore, ATG5 can reportedly interfere with the maintenance of autoreactive T and B cell clones and their imbalanced crosstalk^26–31^, and ATG5 is also implicated in the pathogenesis of RA^32,33^. These studies strongly implicate autophagy as a core mechanism of autoimmunity rather than as a bystander.

One study in dry eye murine model indicated enhanced autophagy in the lacrimal glands and assumed that DE stress activates the autophagy process via hypoxia caused by the loss of lacrimal gland vasculature^10^. However, in our data from non-SS DE patients, autophagy markers from tears and conjunctiva did not significantly increase compared to healthy controls even though we could not directly examine the patient’s lacrimal gland. Our data of NOD/LtJ mice SS animal model showed that protein abundance of ATG5 and LC3B-II/I and their stained feature in lacrimal glands were at 8 weeks of age and became more prominent at 16 weeks of age. Because it has been reported that the salivary and lacrimal gland infiltration occurs at approximately 12 to 16 weeks of age, followed by secretory dysfunction by 16 weeks of age^34^, the increase of autophagy markers coincides timely to the pathogenic course of SS. This temporal coincidence suggests that enhanced autophagy is closely related to autoimmune inflammation and gland dysfunction of SS, not just as a predecessor or by consequence of DE stress as suggested in the experimental DE model.

When we observed that elevated ATG in SS DE was associated with DE clinical parameters, ATG5 expressions in tear and conjunctival epithelium proved to have positive correlations with corneal and conjunctival staining scores. As ocular staining scores showed higher multi-criteria decision analysis weights and higher importance of variable than the Schirmer test, which is a classic ocular criteria in the new diagnostic criteria for primary SS^35^, positive correlations of ATG5 with ocular staining scores are very meaningful. In regards to associations with the Schirmer test, ATG5 in the conjunctival epithelium did not correlate with this value but ATG5 in tears showed weak negative correlations, even though it was not statistically significant. We think very low Schirmer numeric value (2.72 ± 1.88 mm) in SS DE could not reach statistical significant correlation results.

Our data suggests the possibility of ATG5 as a diagnostic and therapeutic marker in SS DE. ATG5 in tear and conjunctival epithelium were upregulated in SS DE but not in non-SS DE compared to healthy controls. Previous studies demonstrated elevated tear cytokines such as IL-6, IL-17 or TNF-α in SS DE but these cytokines are not specific to SS DE because they are also elevated in non-SS DE^36,37^. Although data from a larger clinical study are warranted, ATG5 is expected to serve as a disease-specific diagnostic marker of SS DE. Of the interest, the elevated autophagy markers in tear and conjunctival epithelium were reduced after topical corticosteroid treatment, in conjunction with improvement of clinical parameters in SS DE. The effects of topical corticosteroids on improvement of symptoms and signs in SS DE were very well established^38,39^. Previous studies exhibited immune activation of the conjunctival epithelium and accompanying elevations of cytokines in SS DE^40,41^. Topical corticosteroids may exert its therapeutic effects by inhibiting the immune response and the production of cytokines on the conjunctival epithelium resulting in reduction of autophagy markers in conjunctival epithelium after corticosteroid treatment. Elevated autophagy markers in tears were also reduced after corticosteroid treatment in our study. There is no evidence that the topically applied anti-inflammatory treatment influences lacrimal glands by direct drug delivery. Since lacrimal secretion is diluted with secretions from meibomian glands and ocular surface (cornea and conjunctiva) epithelial cells while constituting the tear film at the ocular surface^42^, elevated autophagy markers in tears in our study might originate from lacrimal glands, as well as ocular surface epithelial cells. Therefore, reduction of autophagy markers in tear after corticosteroid treatment might reflect the therapeutic effects of corticosteroid on conjunctiva epithelium. Also, it should be considered that normalization of neural feedback mechanism to stimulate lacrimal secretion, which is suppressed by ocular surface inflammation^43^, has led to tear recovery and increase of Schirmer values after corticosteroid treatment.

In conclusion, this cross-sectional case-control study demonstrated that autophagy markers in tears and the conjunctival epithelium were upregulated in patients with SS DE, and ATG5 expression positively correlated with the corneal and conjunctival staining scores in SS DE. Additionally, anti-inflammatory treatment with a topical corticosteroid attenuated these increases in autophagy markers, which was accompanied by improved clinical parameters in patients with SS DE. Further studies using SS animal models and large numbers of human samples are required to reveal the role of autophagy on the pathogenesis of SS DE and validate the sensitivity and specificity of ATG5 as a potential biomarker of SS DE.

## Methods

### Study Design

The current study was a cross-sectional case-control research performed at Seoul St. Mary’s Hospital, the Catholic University of Korea in the Department of Ophthalmology. Informed consent was attained from all subjects in this research. The study protocols in this research was designed referencing the Declaration of Helsinki guidelines and was affirmed by the Institutional Review Board of Seoul St. Mary’s Hospital.

### Subjects

Eighty-two female subjects were included in the present study: 40 SS DE patients (78 eyes), 24 non-SS DE patients (48 eyes), and 16 normal healthy subjects (25 eyes) as control. Sixteen of 40 SS DE patients received topical corticosteroid treatment (4 times a day for 1 month, fluorometholone 0.1%, Sam-Il Pharmaceutics Co. Seoul, Korea). All DE patients had disease severity above grade 2, a classification that was used by referencing the severity grading scheme in the International Dry Eye WorkShop (DEWS)^1^. All SS DE patients were confirmed with primary SS from a diagnosis by a rheumatologist according to the 2012 Sjögren International Collaborative Clinical Alliance (SICCA) classification criteria^44^. Patients with secondary SS or subjects taking any disease-modifying medications were excluded from this study. Only females were included in this current study, reflecting on the higher prevalence of SS in females(F:M = 9:1)^45^. The patients with the conditions that could affect dry eye parameters were excluded from this study. They included those with acute or chronic allergic conjunctivitis, lid abnormalities, any ocular surgeries within a span of the last 6 months, contact lens wearers, punctual plug, meibomian gland dysfunction above grade 2, and subjects taking systemic medications and using any topical treatments other than artificial tears within the last 3 months^46^.

### Clinical Assessments

The same investigator (S.-H.C.) assessed the ocular surface and measured the clinical parameters including the Schirmer I test, ocular surface disease index (OSDI) questionnaire ocular surface staining (OSS) scores, and tear film breakup time (TBUT). Standardized Schirmer strips (Eagle Vision, Memphis, TN, USA) were situated in the lateral 1/3 of the lower lid without anesthesia, and after 5 minutes, the length of strips that were wetted was measured. The strips were then frozen and stored for future analysis by cutting them lengthwise in cryotubes. A fluorescein dye (Haag-Streit, Koeniz, Switzerland) was applied to patients and the TBUT was measured by a slit lamp under a cobalt blue light. The average score of three TBUT measurements were used^47^. The ocular staining scores (OSS) including corneal staining scores and conjunctival staining scores were determined according to the SICCA registry ocular examination protocol^48^. To calculate the corneal staining scores, corneal surface images were taken as a screenshot immediately after measuring TBUT. Corneal punctate epithelial erosions (PEEs) were counted and scored that stain with fluorescein. The corneal score was 0 if PEEs were absent, and the score was 1 when 1–5 PEEs could be observed. Additionally, a score of 2 was given to 6–30 PEEs observed, and a 3 if more than 30 PEEs were visible. In the case of more than one patches that could be seen of confluent staining, pupillary area of the cornea, or filament anywhere on the cornea that was stained, an additional point was calculated. The maximum obtainable score for each cornea was 6. To measure the conjunctival staining scores, 1% Lissamine green dye (Leiter’s Pharmacy San Jose, CA, USA) was administered in the inferior conjunctival fornix one drop each in both eyes after fluorescein was washed out with nonpreserved saline solution. After several blinks, the conjunctivae staining scores were examined separately in the temporal and nasal bulbar conjunctivae. A score of 0 was represented 0 to 9 dots for Lissamine green staining; a score of 1 was appointed for 10 to 32 dots, a score of 2 with 33 to 100 dots, and a score 3 indicated >100 dots. Therefore, the maximum possible score was 6 for the conjunctiva (temporal plus nasal).

### Tear Collection and Western Blot Analysis

Tears were collected from patients by Schirmer strips that were tear-soaked, in accordance with the Posa’s protocol^49^. In order for the extraction of the tear fluid, extraction buffer (NaCl 0.5 M, Tween-20 0.5% in 0.1 M phosphate buffered solution) was added to Eppendorf tubes containing Schirmer strips.After one hour, a puncture was made at the bottom of an 0.5 mL tube and the Schirmer was secured inside. The 0.5 mL tube was then transferred into a 1.5 mL tube for centrifugation at 12,000 rpm for 15 min. The gathered tear fluid from the strip was mixed in a sample-loading buffer, boiled for 10 min, and centrifuged. BCA Protein Assay kit (Thermo Fisher Scientific, Rockford, IL, USA) was used for the quantification of the proteins in the sample lysates. Samples buffer with equal amount of protein were separated by 15% SDS-polyacrylamide gel electrophoresis (SDS-PAGE) under reducing conditions and electro-transferred to a PVDF membrane (Millipore, Billerica, MA, USA). PBS with 5% nonfat milk and 0.1% Tween-20 was used for blocking the membrane and incubated at 4 °C for 18 h with primary rabbit polyclonal antibodies targeting LC3B-I and II (1:400, Cell Signaling Technology, Boston, MA, USA) or ATG5 (1:400, Novus Biologicals, Littleton, CO). With the completion of three washes, anti-rabbit horseradish peroxidase-conjugated secondary antibodies (1:10,000, Thermo Fisher Scientific) were used for membrane incubation at RT for 1 h. With another wash 3 times, enhanced chemiluminiscence reagent (ECL; Amersham Biosciences, Sweden) was used to detect protein bands. After, all the membranes underwent a stripping and reprobing process with mouse monoclonal anti-β-actin antibody (Santa Cruz Biotechnology, Dallas, TX, USA) to provide a normalizing reference. Each experiment normalized the relative expression levels by image analysis by including a stain for β-actin. We performed 4–6 independent experiments.

### Impression Cytology

We performed impression cytology (IC) at least 15 min after all ocular examination. Polyethersulfone filters (Suopor 200 membrane, Pall Corporation, Port Washington, NY, USA) were used by cutting into two halves (13 × 6.5 mm) and applying them to both the superior-nasal and superior-temporal non-exposed bulbar conjunctiva. The filter samples of the superior-temporal conjunctivas were placed in tubes that contained 2 ml of PBS with 0.05% paraformaldehyde (PFA) to be used for immunostaining. Filters from the superior-nasal conjunctivas were used for RNA extraction within 3 hours of their sampling.

### Immunofluorescent Staining

IC filter papers with the detached epithelial cells were firmly pressed to silane-coated slides (Muto Pure Chemicals co., ltd. Tokyo, Japan), and epithelial cells transferred to the slides were used for immunofluorescent staining according to Baudouin’s protocol^50^. Briefly, cold methanol was used to fix the cells and 0.1% Triton X-100 was applied for cell permeabilization. Then, nonspecific reactions were blocked by incubation with 10% goat serum for 1 h. After, they were then incubated with anti- LC3B-I, II or ATG5 antibody (1:400) and washed twice with PBS. Finally they were incubated with goat Alexa Flour 488–conjugated anti-rabbit IgG Ab (1:400, ThermoFisher scientific, Waltham, MA, USA) in PBS and the stain was captured by confocal microscopy (LSM 510 Meta (Carl Zeiss Meditec Inc. Dublin, CA) and transferred to Photoshop (Adobe systems, Santa Clara, CA).

### RNA Isolation and Real-Time PCR

The RNA from IC was isolated by TRIzol reagent (Gibco-Invitrogen, Grand Island, NY, USA) according to a modified protocol^51^. SuperScript III^TM^ reverse transcriptase (Invitrogen) was used with random hexamers to synthesize the first strand of complementary DNA (cDNA) and a SYBR Green I real-time PCR method was followed. The 2^−ΔΔCt^ was calculated for relative quantification and for internal calibration GAPDH was used. The primers used in this study were LC3B-II (Forward: 5′-CTT TGG GTG CGA CTT GAC G-3′, Reverse: 5′-GTC GAC CCC GCT CCT TTT-3′) and ATG5 (Forward: 5′-AGG AGA GCC TGT ACC TAT GGA-3′, Reverse: 5′-TTC TCT GTT GCG CTT TTC TGA-3′).

### Animals

Because a human lacrimal gland biopsy is a relatively invasive procedure, we evaluated the expression levels of ATG5 and LC3B-II from the lacrimal gland of NOD/LtJ mice, a SS animal model. Lacrimal glands of NOD/LtJ was provided by Prof. Mi-La Cho (The Catholic University of Korea, Seoul, Korea) and breeding pairs of NOD/LtJ mice were originally acquired from Charles River Laboratory (Wilmington, MA, USA). The NOD mouse is a widely used model for diabetes mellitus type I, but also for SS. It has been reported that lymphocytic infiltrates in NOD mice were detected in the exocrine glands in addition to the pancreas, resulting in sialadenitis and dacryoadenitis^34^, and also several major autoantibodies (ANA, anti-SSA/Ro, anti-SSB/La) in common with SS patients were exhibited^52^. BALB/c mice (Oriental Bio, Inc., Korea) were used for control animal. Both lacrimal glands were removed from six mice of each 8- and 16-week-old NOD/LtJ, and 16-week-old BALB/c mouse. One of lacrimal glands was immediately frozen for western plot. Western blot analysis and immunostaining of ATG5 and LC3B-II were performed as described above. The other of lacrimal glands was fixed in 10% formalin solution for overnight at room temperature. Samples were then immersed in OCT embedding compound (Tissue-Tek, Sakura, Osaka, Japan) and frozen at −80 °C. Frozen samples were cut in 6-μm-thick sections using a cryostat. The subsequent immunostaining process was as described above. Animal protocols in this current experiment were approved by the Institutional Animal Care and Use Committee (IACUC) at the College of Medicine, The Catholic University of Korea. Additionally, all procedures performed in animal studies were in alignment with the standards for the Use of Animals in Ophthalmic and Vision Research in the Association for Research in Vision and Ophthalmology Statement.

### Statistical Analysis

All descriptive data in this study were analyzed with the SPSS software (version 15.0, SPSS Inc, Chicago, IL) and GraphPad Prism (version 5.0, GraphPad Inc. La Jolla, CA), and are presented as the mean and SD. *Shapiro-Wilk* test was utilized to assess the normality of the data, while group comparisons were analyzed by *One-way analysis of variance (ANOVA*) and the *t*-test. *Pearson* correlation coefficients between autophagy markers and clinical parameters were calculated to explore the relationships between the expression level of autophagy markers and clinical variables. Two-sided *P* values < 0.05 were considered to be significant.

## Electronic supplementary material

Supplementary Information
