# Supplementary material for: Elevation of autophagy markers in Sjögren syndrome dry eye
Source: Sci Rep. 2017 Dec 8;7:17280. doi: 10.1038/s41598-017-17128-0 (PMC5722946; doi:10.1038/s41598-017-17128-0)

SREP-17-15868C

Elevation of autophagy markers in Sjögren syndrome dry eye

Yong-Soo Byun,1,2,+ Hyun-Jung Lee,2,+ Soojung Shin2, So-Hyang Chung1,2,*

1Department of Ophthalmology and Visual Science, Seoul St. Mary's Hospital, Catholic University of Korea, College of Medicine, Seoul, Republic of Korea
2Catholic Institute for Visual Science, Catholic University of Korea, College of Medicine, Seoul, Republic of Korea

Fig1A

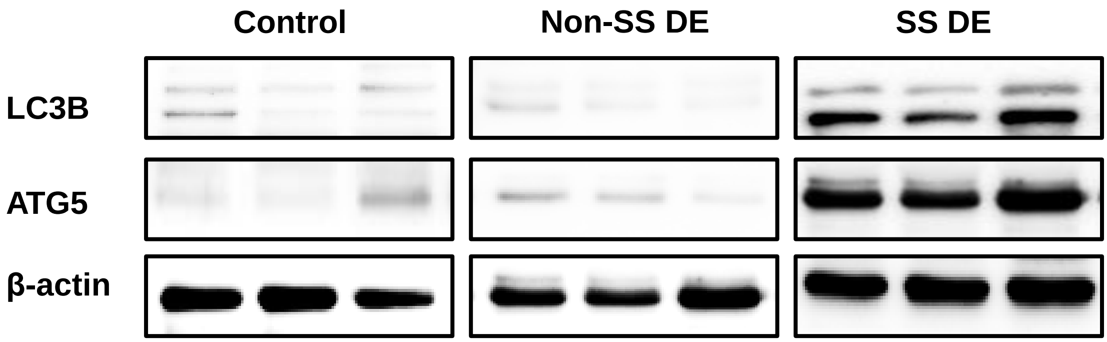

Fig1A

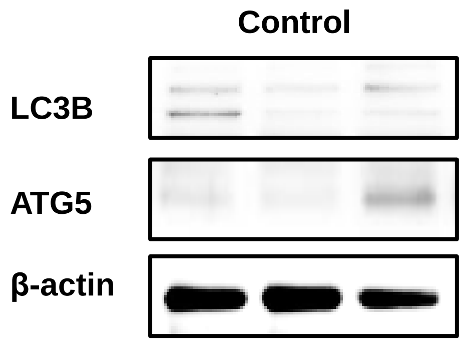

Since the molecular weight of each antibody is different, there are three developed films cut from one original gel, as shown below.

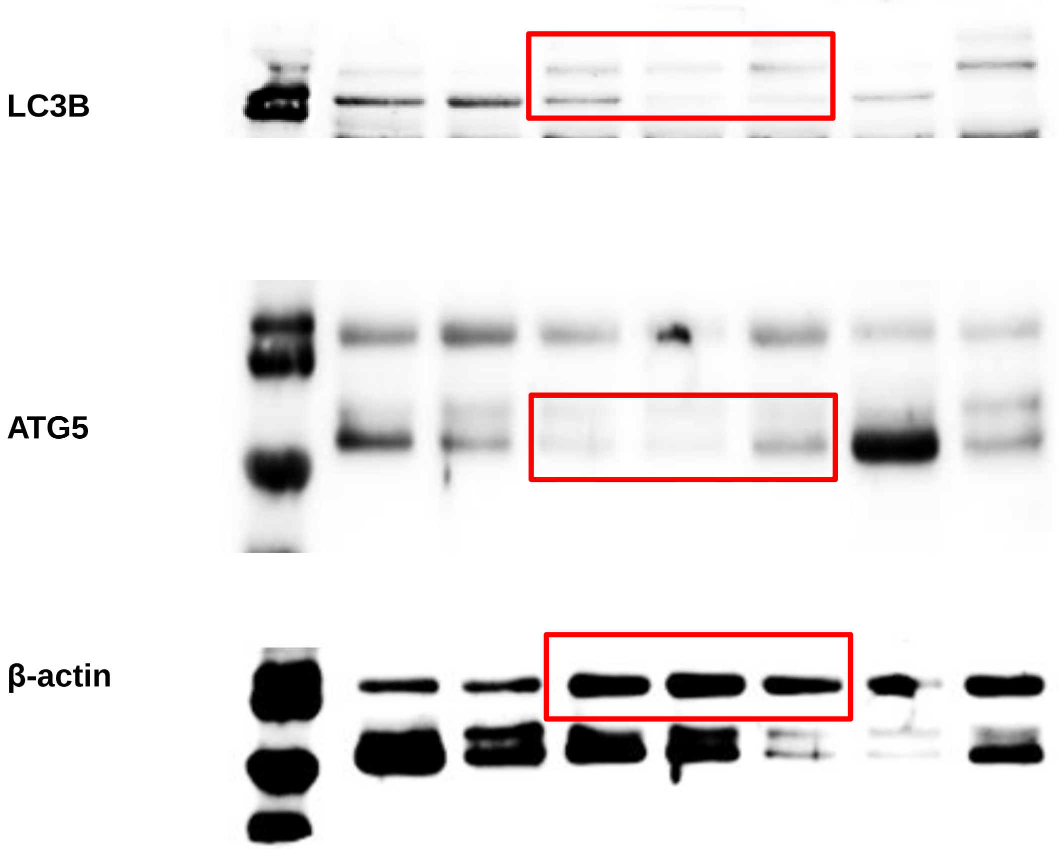

Fig1A

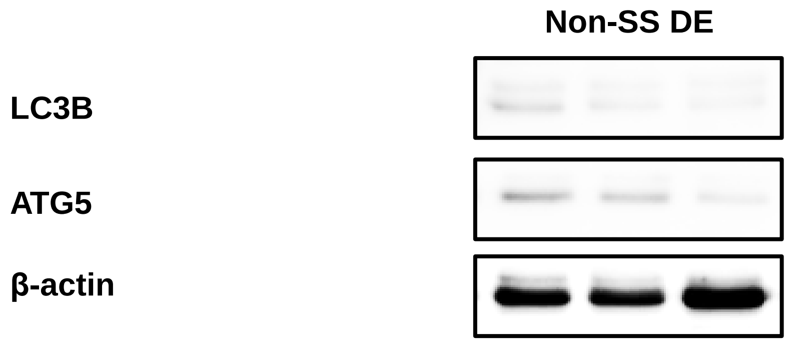

Since the molecular weight of each antibody is different, there are three developed films cut from one original gel, as shown below.

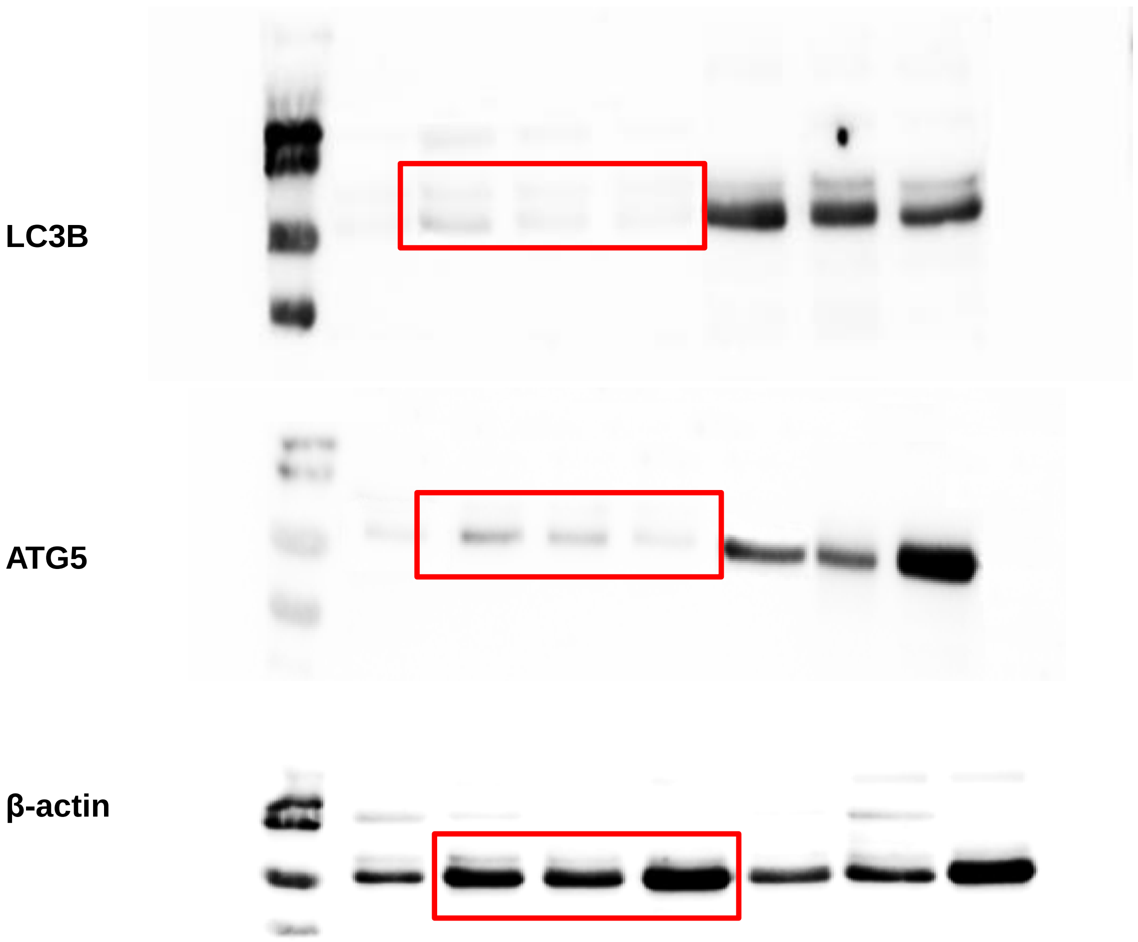

Fig1A

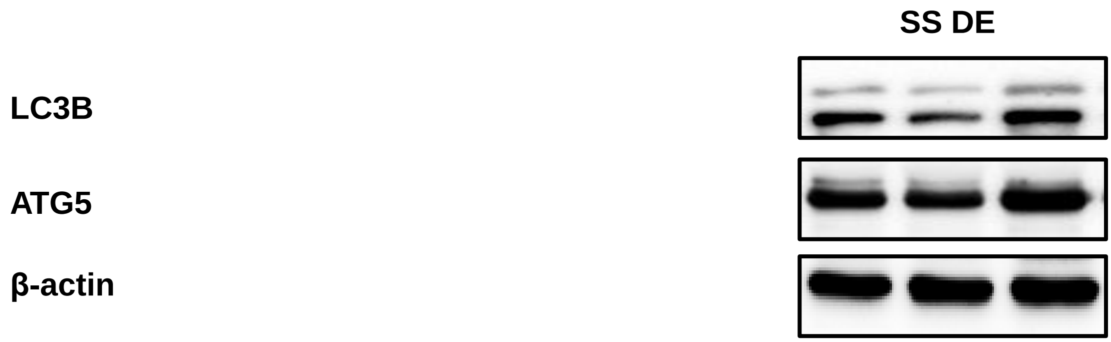

Since the molecular weight of each antibody is different, there are three developed films cut from one original gel, as shown below.

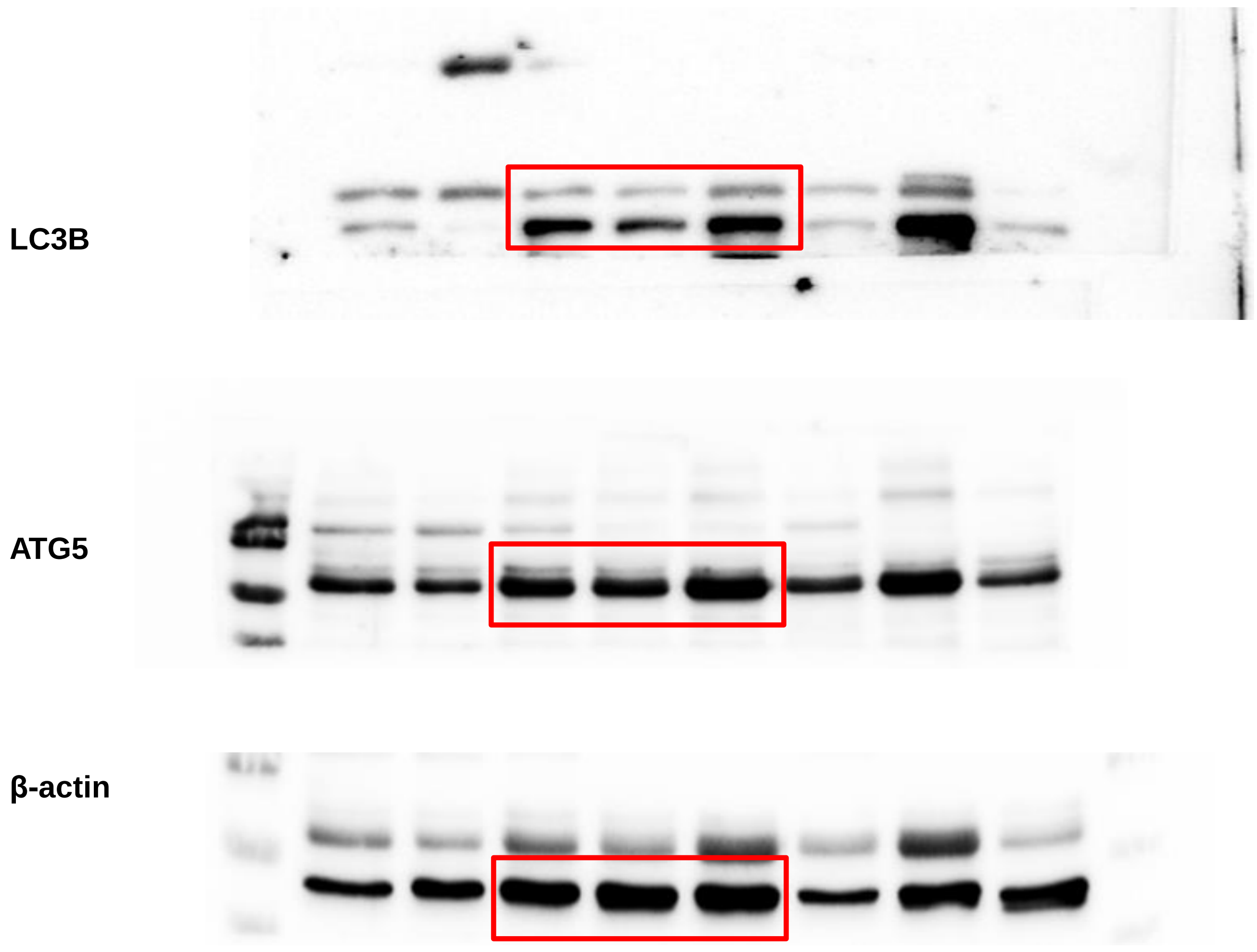

Fig2B

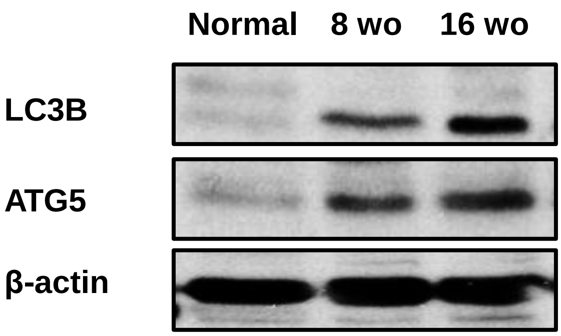

Fig2B

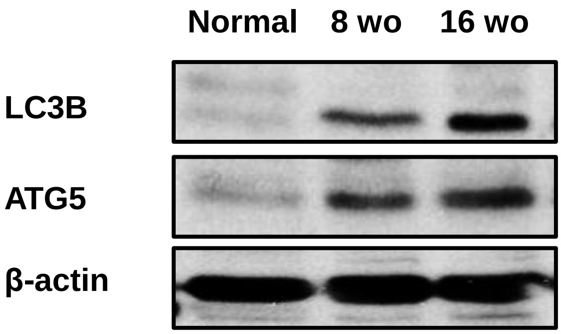

Since the molecular weight of each antibody is different, there are three developed films cut from one original gel, as shown below.

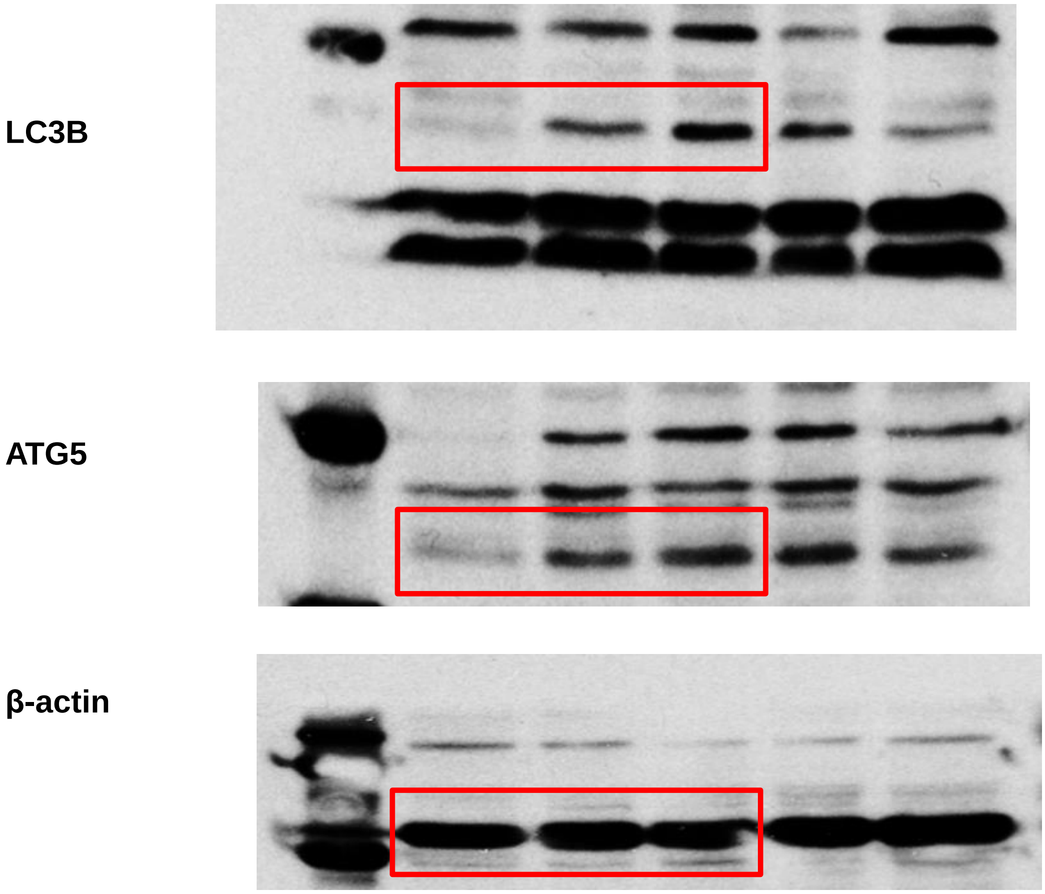

Fig4A

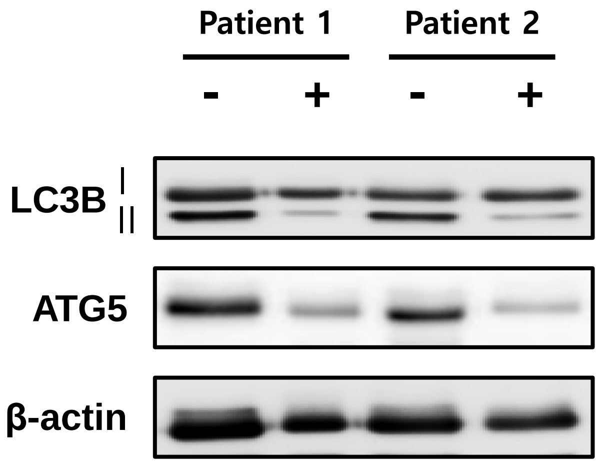

Fig4A

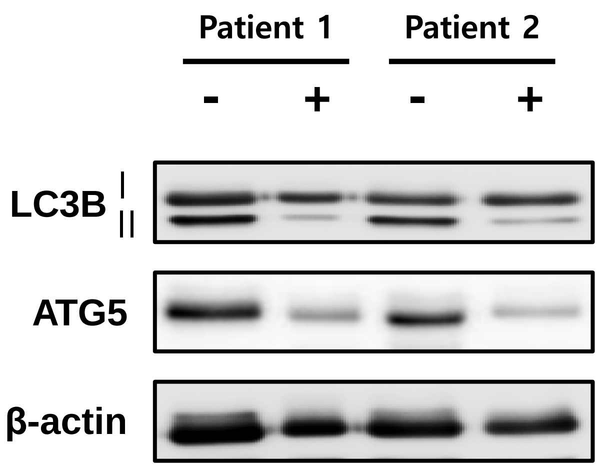

Since the molecular weight of each antibody is different, there are three developed films cut from one original gel, as shown below.

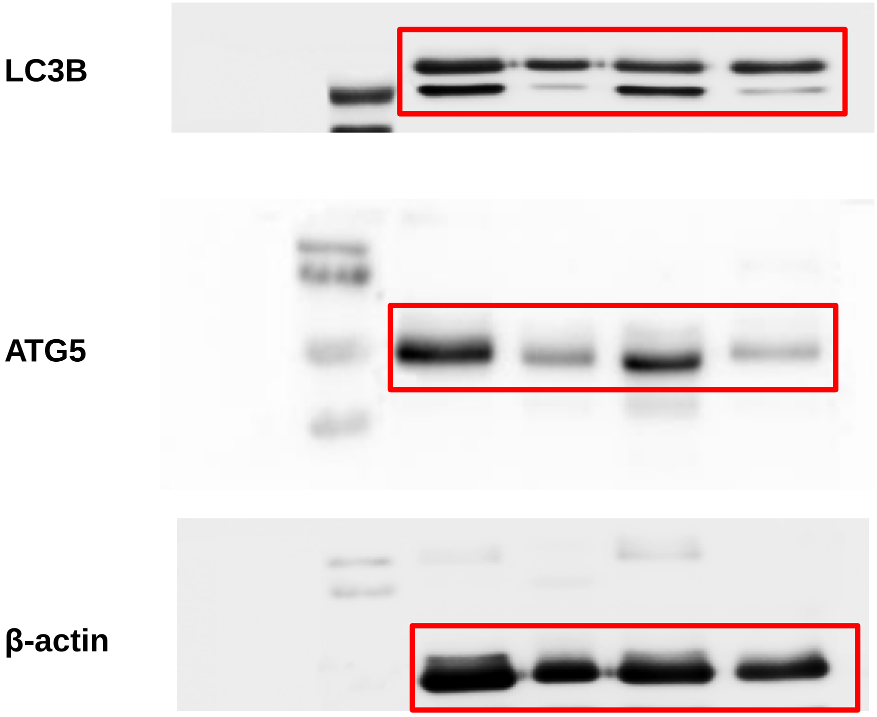

Supplement: Supplementary file 1 — Supplementary Information [file 41598_2017_17128_MOESM1_ESM.pdf]
